# Exploring Heterogeneity in Vestibular Migraine Using Individualized Differential Structural Covariance Network Analysis

**DOI:** 10.1111/cns.70599

**Published:** 2025-09-08

**Authors:** Wen Chen, Hongru Zhao, Lingling Dai, Xing Xiong, Qifang Feng, Jun Ke, Chunhong Hu

**Affiliations:** ^1^ Department of Radiology The First Affiliated Hospital of Soochow University Suzhou Jiangsu China; ^2^ Institute of Medical Imaging Soochow University Soochow Jiangsu Province People's Republic of China; ^3^ Department of Neurology The First Affiliated Hospital of Soochow University Suzhou Jiangsu China

**Keywords:** gray matter, heterogeneity, individualized analysis, magnetic resonance imaging, structural covariance network, vestibular migraine

## Abstract

**Background:**

The high heterogeneity in vestibular migraine (VM) complicates understanding its precise pathophysiological mechanisms and identifying potential biomarkers. This study investigated the heterogeneity in VM using a newly proposed method called Individualized Differential Structural Covariance Network (IDSCN) analysis.

**Methods:**

Structural T1‐weighted MRI scans were performed on 55 patients with VM and 65 healthy controls, and an IDSCN was constructed for each patient. We studied the extent of heterogeneity in the IDSCNs, summarized the distribution of differential edges, and clustered the patients into subtypes with the shared differential edges. Imaging–clinical association analyses were conducted on both the subtype classification and the differential edges exhibiting significant inter‐subtype differences.

**Results:**

Patients with VM demonstrated notable heterogeneity in the number of significantly altered IDSCN edges, while sharing several common differential connections that were mainly distributed among the parietal, subcortical, and cerebellar regions. Two robust and distinct neuroanatomical subtypes of VM were identified, which were associated with headache frequency. The differential edge between the left paracentral lobule and right pallidum was associated with both headache frequency and occurrence.

**Conclusions:**

These findings indicate the importance of considering individual differences in VM research and may offer insights for precise diagnosis and individualized treatment of the disease.

## Graphical abstract

This study based on individualized differential structural covariance network analysis revealed that patients with vestibular migraine (VM) had considerable heterogeneity in structural covariance aberrance and could be categorized into two distinct neuroanatomical subtypes associated with headache frequency. The findings indicate the importance of considering individual differences in VM research and may offer insights for precise diagnosis and individualized treatment of the disease.

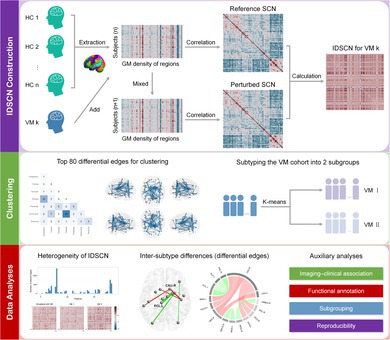

## 1 Introduction

As a heterogeneous and disabling disorder, vestibular migraine (VM) has diverse clinical presentations primarily reflected as recurrent episodes of vertigo, which are often but not always accompanied by headaches [1, 2]. With a lifetime prevalence of 1%–3% in the general population, this disease is the leading cause of episodic dizziness in adults [3, 4]. Despite its relatively high prevalence and substantial economic burden [4], VM remains underdiagnosed, and its pathophysiology is poorly understood [5, 6]. Understanding neural mechanisms and identifying potential biomarkers may improve diagnostic and therapeutic strategies for VM.

In recent years, the evolution of neuroimaging techniques has substantially improved our insights into the neural basis of VM. Existing research employing resting‐state functional MRI (rs‐fMRI) has collectively revealed aberrance of the brain functional network related to multisensory integration and emotional and cognitive regulation in patients with VM [7, 8, 9, 10, 11]. However, the structural foundation of the observed functional network alterations requires further elucidation. The structural covariance network (SCN), a type of network designed to depict the covariation in morphology between brain regions, reflects anatomical connectivity, mutually trophic effects, and common experience‐driven plasticity [12, 13, 14, 15]. Unlike the commonly employed functional connectivity, structural covariance captures brain connectivity characteristics over a longer timescale and represents more stable characteristics [16]. The SCN can be reshaped in disease states [12], making it a valuable tool for investigating the neural mechanisms underlying various neurological disorders.

Nonetheless, conventional case–control studies on SCN have only addressed differences at the group level (i.e., the “average patient”) while ignoring heterogeneity among individuals [17]. Interindividual heterogeneity is a major contributor to inconsistent findings and hinders the development of dependable neuroimaging markers for clinical applications [18]. Structural voxel‐based morphometry investigations of VM have yielded highly inconsistent and conflicting results [19, 20, 21, 22]. Moreover, our prior study indicated specific variations in the altered topological properties of gray matter (GM) morphological networks in patients with VM [23]. Considering the heterogeneity presented in both the clinical profile and neuroimaging findings of VM, it is necessary to address individual‐level differences in SCN to better understand the neurobiological substrates underlying the disease.

An increasing number of research studies have acknowledged the significance of heterogeneity‐related individual‐level differences and has focused on individual‐level neuroimaging aberrances in neurological disorders [24]. In particular, Liu et al. [25] developed an individualized differential structural covariance network (IDSCN) analysis method that facilitates the identification of differential structural covariance on an individual basis. Using this approach, they successfully identified two neuroanatomical subtypes of schizophrenia with different symptom patterns, which effectively addressed the clinical and biological heterogeneity of the disorder [25]. Subsequently, this novel methodology has been effectively used to resolve heterogeneity in various neurological diseases [26, 27, 28, 29, 30]. Consequently, we hypothesized that distinct neuroanatomical subtypes of VM with potential differences in clinical profiles could be revealed by IDSCN.

**FIGURE 1:**
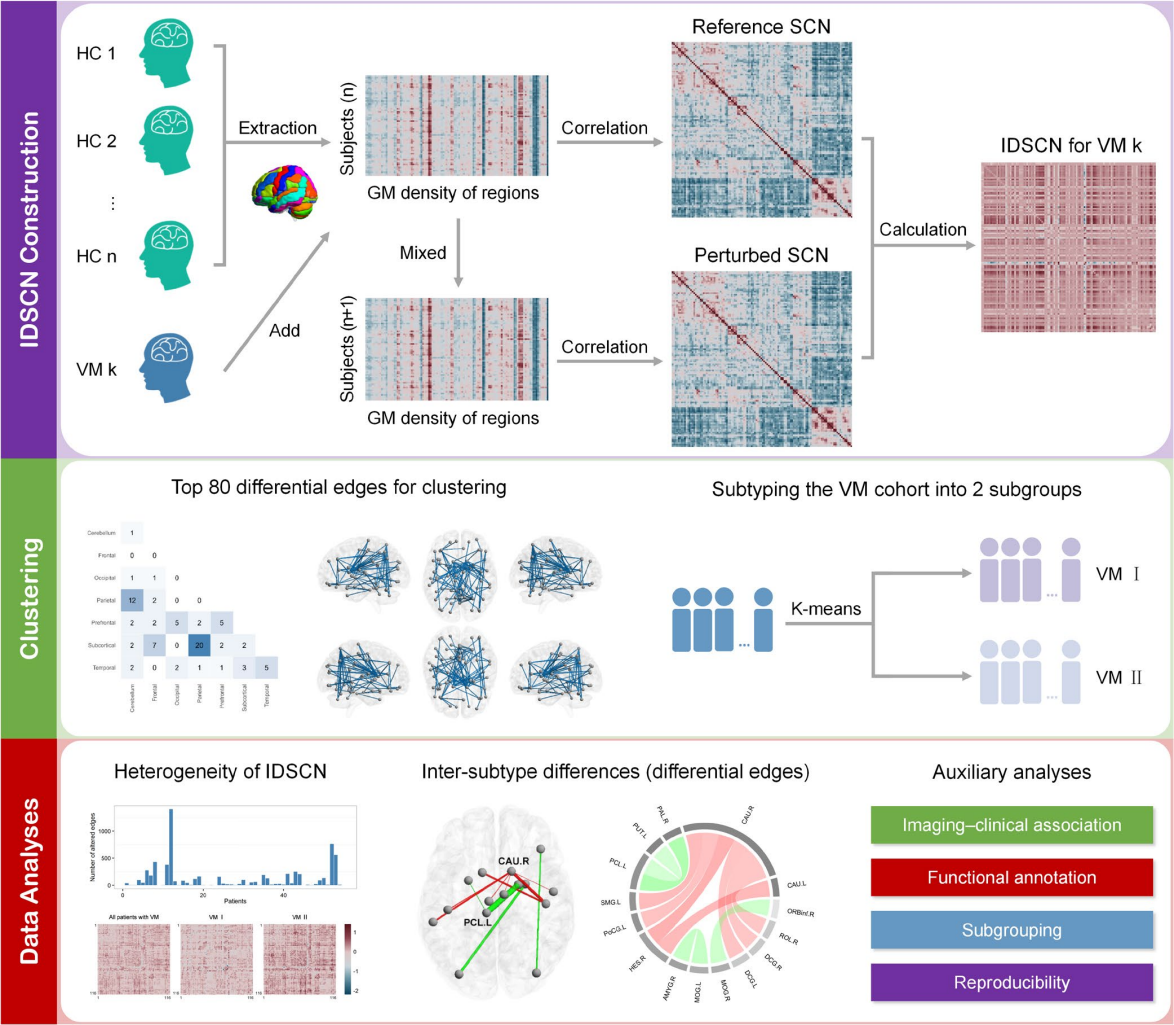
Summary of the study design and analysis pipeline. (1) The top purple panel shows the IDSCN construction workflow. A reference SCN was initially built using the GM density data from all controls. The integration of VM k into the HC group generated a new SCN, known as the perturbed network. IDSCN for VM k was determined by calculating the Z‐score of the difference between the perturbed network and reference network. (2) The middle green panel shows the clustering process. The VM cohort was clustered into two subgroups (i.e., VM I and II) using the k‐means method, with the top 80 differential edges treated as features. (3) The bottom red panel illustrates the data analysis procedures, including investigations on the heterogeneity of IDSCN, inter‐subtype differences in differential edges, and auxiliary analyses (i.e., imaging–clinical association, functional annotation, subgrouping, and reproducibility analyses). GM, gray matter; HC, healthy control; IDSCN, individualized differential structural covariance network; SCN, structural covariance network; VM, vestibular migraine; VM I, subtype 1 of VM; VM II, subtype 2 of VM.

We tested our hypothesis by employing IDSCN to delineate individual‐level differential structural covariance aberrances in VM. Our study was structured as follows: First, we explored the extent of heterogeneity in the IDSCNs of patients with VM. Considering the observed clinical and neuroimaging heterogeneity, we expected high heterogeneity in the VM cohort. Second, we obtained the significantly altered structural covariance edges for each patient and summarized the distribution of differential edges across patients. Third, we utilized the shared differential edges to cluster patients with VM into distinct subtypes and examined the neuroimaging and clinical profiles of the subtypes (Figure 1).

## 2 Materials and Methods

### 2.1 Participants

This study was approved by the Ethics Committee of the First Affiliated Hospital of Soochow University. All participants provided written informed consent before entering this research. Fifty‐five right‐handed patients diagnosed with VM [31, 32] were enrolled from the vertigo and migraine outpatient center of our hospital. Comprehensive evaluations, including videonystagmography, vestibular caloric test, video head impulse test, and audiometry tests, were performed to exclude peripheral vestibular diseases. For all patients, demographic and clinical information was systematically recorded via a standardized questionnaire. The collected data included sex, age, education level, migraine disease duration, vertigo disease duration, headache frequency (days per month), 10‐point Visual Analog Scale (VAS), Dizziness Handicap Inventory (DHI), Migraine Disability Assessment Scale (MIDAS), Headache Impact Test‐6 (HIT‐6), Patient Health Questionnaire‐9 (PHQ‐9), and Generalized Anxiety Disorder‐7 (GAD‐7). We also documented the headache occurrence for each patient, classifying them as either having or not having a painful phase. All included patients were in the interictal period, defined as being free of both migraine and vertigo attacks for at least 3 days before and 1 day after the MRI acquisition. Furthermore, none of the patients had used any prophylactic medication, nor were they undergoing treatment at the time of the study. Some had used therapeutic medications in the past but did not take any therapeutic medication within 3 days prior to the MRI scan to minimize their potential impact on the results.

Additionally, 65 healthy controls (HCs) matched for sex, age, and education level were included. These controls had no prior personal or family history of migraine, vertigo, or any other primary headache diseases, as per established diagnostic guidelines. The exclusion criteria for all participants included left‐handedness, other neurological or mental disorders, other pain conditions, drug or alcohol abuse, and contraindications to MRI. Left‐handed participants were excluded due to known differences in hemispheric lateralization of vestibular function (i.e., right‐handers typically exhibit right‐hemispheric dominance while left‐handers exhibit left‐hemispheric dominance) [33]. Given the relatively low prevalence of left‐handedness and its potential effect on the localization of vestibular processing cortices, excluding left‐handed participants helps reduce confounds and enhance sample homogeneity.

### 2.2 MRI Acquisition

Details regarding MRI scanning have been documented in our previous study [23]. Briefly, a 3.0‐Tesla MRI system (MAGNETOM Skyra, Siemens Healthcare, Erlangen, Germany) was employed to obtain high‐resolution T1‐weighted anatomic images from all participants. Herein, only the T1‐weighted MRI sequence was used, as the construction and analysis of the IDSCN rely solely on the structural anatomical information provided by T1 images. The scanning parameters were as follows: repetition time = 2300 ms, echo time = 2.98 ms, field of view = 256 × 256 mm^2^, matrix = 256 × 256, slice thickness = 1 mm, and slice number = 192.

### 2.3 Data Preprocessing

T1 image preprocessing was conducted using the Computational Anatomy Toolbox (CAT12, http://www.neuro.uni‐jena.de/cat/). The main steps involved bias‐field correction, segmentation, and normalization (resampling to 1.5 × 1.5 × 1.5 mm^3^). More details regarding these procedures can be found in our prior publication [23]. The GM density map and total intracranial volume (TIV) of each participant were finally utilized in subsequent analyses.

### 2.4 Construction of the IDSCN

We constructed an IDSCN for each VM subject based on the methodology proposed by Liu et al. [25]. Specifically, the process included the following steps [25, 26]: (1) The brain was segmented into 116 regions based on the Automatic Anatomical Labeling (AAL) atlas, and the average GM density from each region was extracted for each HC subject, resulting in an *n* × 116 matrix (where *n* denotes the number of HC subjects). (2) Partial correlations, controlling for sex, age, education level, and TIV, were computed for this matrix to generate a 116 × 116 reference SCN (rSCN). (3) The average signal extracted from the brain region of VM patient *k* was incorporated to the signals of the HC group, yielding a new (*n* + 1) × 116 matrix. (4) The perturbed network (pSCN), whose size was 116 × 116 (same with the rSCN), was acquired in accordance with the second step. (5) The difference (ΔSCN) between pSCN and rSCN was computed as ΔSCN = pSCN—rSCN. (6) The IDSCN for VM patient *k* was constructed using the Z‐score of ΔSCN, which was calculated according to the following formula:

$$Z=\frac{\text{ΔSCN}}{\left(1-{\text{rSCN}}^{2}\right)/\left(n-1\right)}$$

By repeating steps (3) to (6), an IDSCN was ultimately obtained for each individual with VM, comprising 6670 edges connecting 116 distinct regions.

### 2.5 Subtypes of VM Identified by IDSCN

The *Z*‐values of the edges in the IDSCN were subsequently converted into *p*‐values. False discovery rate (FDR) correction at *p* < 0.05 was employed to select those significantly altered edges relative to the HC group from the total 6670 edges. Using the top 80 edges as features, we then applied the *k*‐means algorithm to cluster the VM patients. The optimal cluster number ranging from 2 to 20 was determined by the silhouette value [26], and the k‐means was repeated 100 times [34]. Finally, we clustered the VM patients into two subtypes because the silhouette value was maximized when the number of clusters was set to two.

To validate our findings, we performed two‐sample *t*‐tests controlling for sex, age, education level, and TIV to evaluate whether the identified edges significantly differed between the two subtypes (statistical significance was set at FDR‐corrected *p* [*p*_FDR_] < 0.05). We further investigated whether different subtypes exhibited significant differences in demographics and clinical data. The normality of continuous variables was first assessed using the Shapiro–Wilk test. For comparisons, we then employed the following statistical tests: two‐sample *t*‐tests for normally distributed continuous data, Mann–Whitney *U* tests for non‐normally distributed data, and chi‐square or Fisher's exact tests for categorical variables (statistical significance was set at *p* < 0.05).

### 2.6 Imaging–Clinical Association Analyses

Imaging–clinical association analyses based on multivariate regressions were conducted by using imaging features (neuroanatomical subtypes, and differential edges showing significant inter‐subtype differences) as independent variables, with age, sex, education level, and TIV as controlling variables included alongside the imaging features in the regression models. Dependent variables were divided into two categories: continuous variables (i.e., migraine disease duration, vertigo disease duration, headache frequency, VAS, DHI, MIDAS, HIT‐6, PHQ‐9, and GAD‐7), which were analyzed using multivariate linear regression, and binary variables (i.e., headache occurrence, and history of therapeutic medication use), which were analyzed using multivariate logistic regression. Variance inflation factors (VIF) were calculated to assess multicollinearity, and variables with a VIF greater than 5 were excluded [35]. The normality of residuals in the multiple linear regression models was tested using the Shapiro–Wilk test, and models with non‐normally distributed residuals were discarded from analysis. Imaging features with a *p* < 0.05 were considered significantly associated with the corresponding clinical indices, and only models demonstrating these significant associations were retained for interpretation.

### 2.7 Functional Annotation Analyses

To better understand the functional implications of differential edges exhibiting significant inter‐subtype differences, we conducted functional annotation analyses using the NeuroSynth database [36]. The procedure was carried out with the Brain Annotation Toolbox (BAT), a tool designed to convert voxel‐level functional information from NeuroSynth into network‐level functional annotations [37]. The significance of functional terms associated with the differential edges was assessed through a permutation test. A total of 10,000 permutations were performed, and results with an uncorrected *p* < 0.05 were considered statistically significant.

### 2.8 Subgroup Analyses

We performed exploratory subgroup analyses to evaluate potential differences in (1) the top 80 differential edges and (2) the proportions of subtype 1/subtype 2 classifications across key clinical variables: sex (male vs. female), migraine disease duration (shorter [<median] vs. longer [≥median]), vertigo disease duration (shorter [<median] vs. longer [≥median]), headache occurrence (yes vs. no), and history of therapeutic medication use (yes vs. no). Edge‐wise comparisons were conducted with two‐sample *t*‐tests (statistical significance was set at *p*_FDR_ < 0.05), and subtype proportion differences were assessed using chi‐square tests or Fisher's exact tests when appropriate (statistical significance was set at *p* < 0.05).

### 2.9 Reproducibility Analyses

The following analyses were performed to confirm the robustness of our findings: (1) To rule out the possibility that the subtyping results were influenced by the number of top differential edges (i.e., 80), we reanalyzed the data by adopting distinct numbers of top differential edges (i.e., 60, 70, and 90). The consistency between the subtyping results were assessed using the adjusted Rand index (ARI) [38]. (2) To assess the stability of the significantly different differential edges between subtypes, we reanalyzed the comparisons through nonparametric permutation tests (10,000 iterations) controlling for sex, age, education level, and TIV. Statistical significance was set at *p*_FDR_ < 0.05.

### 2.10 Statistical Analyses on Demographic and Clinical Data

Demographic and clinical data were analyzed using the SPSS software (SPSS 27.0.1 Inc., Chicago, IL). For continuous variables, the normality of the data was first assessed using the Shapiro–Wilk test. Then, two‐sample *t*‐tests (evaluating data with normal distribution) or Mann–Whitney *U* tests (evaluating data not normally distributed) were applied to compare the differences between the VM group and the HC group. For categorical variables, chi‐square tests or Fisher's exact tests were used when appropriate. The statistical significance threshold was set at *p* < 0.05.

## 3 Results

### 3.1 Clinical Demographics

There were no significant differences between the VM and HC groups in terms of age (46.16 ± 11.78 years vs. 48.25 ± 12.37 years; *p* = 0.225, Mann–Whitney U test), sex (8 male/47 female vs. 10 male/55 female; *p* = 0.898, chi‐square test), or education level (10.36 ± 4.79 years vs. 11.72 ± 4.75 years; *p* = 0.169, Mann–Whitney *U* test). The VM group had an average migraine disease duration of 11.81 ± 11.38 years and a vertigo disease duration of 7.15 ± 8.76 years. Headache occurrence was recorded as 50 patients with a painful phase and 5 patients without. The average headache frequency was 2.17 ± 2.36 days per month. Additionally, 27 patients with VM had a history of therapeutic medication use, while 28 patients did not. The average scores of the scales in the VM cohort were as follows: VAS (6.42 ± 2.14), DHI (52.93 ± 16.33), MIDAS (10.97 ± 11.86), HIT‐6 (55.29 ± 10.72), PHQ‐9 (5.87 ± 5.21), and GAD‐7 (4.73 ± 4.05).

### 3.2 Heterogeneity of IDSCN in VM

**FIGURE 2:**
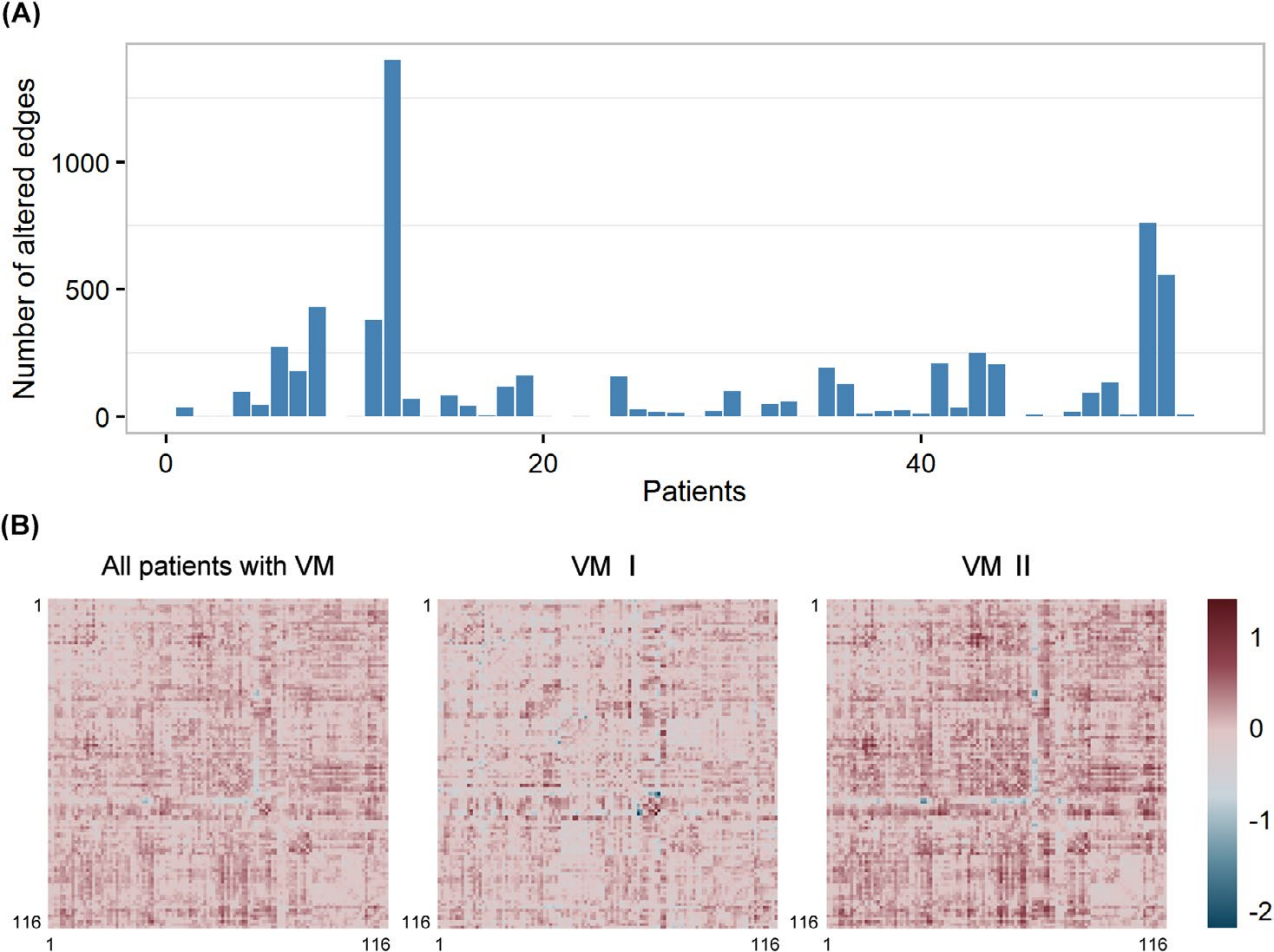
Heterogeneity of IDSCN in VM. (A) In the VM cohort, the number of significantly altered edges exhibited a large variation among patients. (B) The two subtypes of VM had different general patterns of IDSCN. The illustrated general patterns were obtained by calculating the mean maps for each group, where the axes represent the indexes of the brain regions. IDSCN, individualized differential structural covariance network; VM, vestibular migraine; VM I, subtype 1 of VM; VM II, subtype 2 of VM.

We obtained significantly altered structural covariance edges for each patient with VM. Overall, patients with VM had 119 ± 230 significantly altered structural covariance edges, and the number of significantly altered edges exhibited a large variation from patient to patient (Figure 2A). This finding indicates the high heterogeneity of IDSCN among patients with VM.

### 3.3 Distribution of Differential Structural Covariance Edges

The distribution of differential edges across patients was assessed by dividing the top 80 edges into within‐area and between‐area categories. Definitions of the areas were derived from prior studies [39, 40], where the AAL 116 regions were categorized into seven major areas. The number of edges falling within and between these areas is shown in Figure S1. Notably, these edges were predominantly distributed between the parietal lobe and subcortical regions, as well as between the parietal lobe and cerebellum.

### 3.4 Identification of VM Subtypes

**FIGURE 3:**
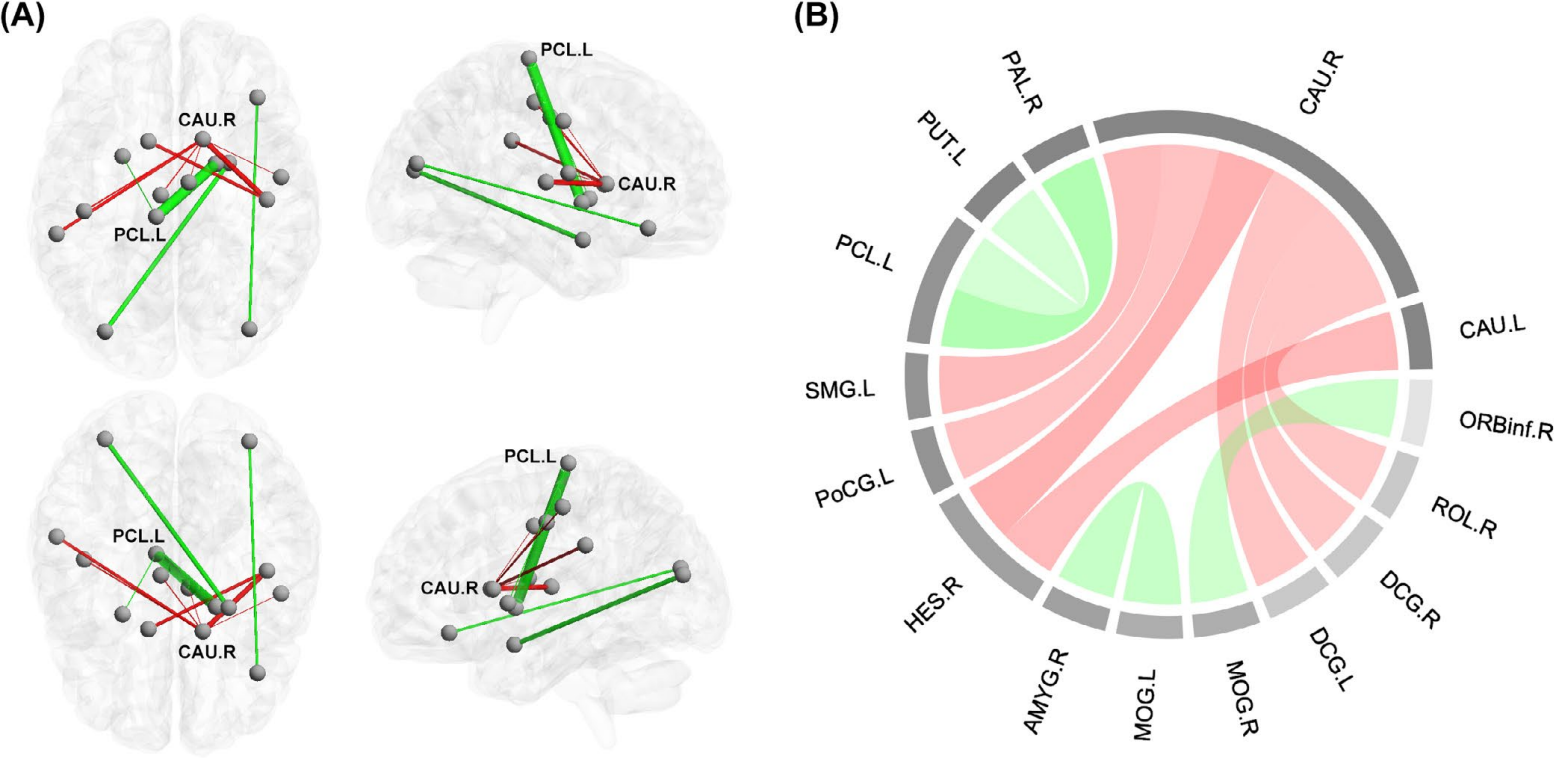
Illustrations of differential edges with significant differences between the two VM subtypes. (A) The identified edges mapped on the Ch2 template can be classified into two classes with opposite directions of change between subtypes. Subtype 1 demonstrated higher *Z*‐scores than subtype 2 in the red edges and lower *Z*‐scores than subtype 2 in the green edges. The thickness of the lines is proportional to the weight of the connections. CAU.R and PCL.L are the core nodes in the identified subnetwork (with a degree of ≥ 2). (B) The connectogram provides a comprehensive representation of the nodes and edges in the subnetwork (red chords: Subtype 1 > subtype 2; green chords: Subtype 1 < subtype 2). Sex, age, education level, and TIV were controlled as confounding covariates. AMYG, amygdala; CAU, caudate; DCG, median cingulate and paracingulate gyri; HES, Heschl gyrus; L, left; MOG, middle occipital gyrus; ORBinf, orbital part of the inferior frontal gyrus; PAL, pallidum; PCL, paracentral lobule; PoCG, postcentral gyrus; PUT, putamen; R, right; ROL, Rolandic operculum; SMG, supramarginal gyrus; TIV, total intracranial volume; VM, vestibular migraine.

Based on the top 80 differential edges, VM patients were clustered into two distinct subtypes (subtype 1, *n* = 19; subtype 2, *n* = 36), which exhibited distinct IDSCN patterns (Figure 2B). Among the top 80 edges of IDSCN, 11 edges demonstrated significant differences between the two VM subtypes after controlling for sex, age, education level, and TIV (*p*_FDR_ < 0.05). These 11 edges fall into two distinct classes, exhibiting opposite directions of change between subtypes. In one class of edges, subtype 1 had higher Z‐scores compared to subtype 2, including edges that connected the caudate (CAU) with the Heschl gyrus (HES), supramarginal gyrus (SMG), postcentral gyrus (PoCG), median cingulate and paracingulate gyri (DCG), and Rolandic operculum (ROL). In the other class of edges, subtype 1 had lower Z‐scores compared to subtype 2, including edges that connected the paracentral lobule (PCL) with the pallidum (PAL) and putamen (PUT), as well as edges that connected the middle occipital gyrus (MOG) with the amygdala (AMYG) and the orbital part of the inferior frontal gyrus (ORBinf) (Table 1 and Figure 3). For the comparison of demographics and clinical data between subtypes, no statistically significant differences were observed in any of the indices (all *p* > 0.05).

**TABLE 1:** Statistical results of differential edges with significant differences between the two subtypes of VM (controlling for sex, age, education level, and TIV).

Edge/condition	*T*‐value	*p*	*p*_FDR_
*Subtype 1 > subtype 2*			
CAU.L—HES.R	3.569	8.140e‐04	**1.580e‐02**
CAU.R—HES.R	3.997	2.157e‐04	**8.628e‐03**
CAU.R—SMG.L	3.505	9.877e‐04	**1.580e‐02**
CAU.R—PoCG.L	3.114	3.083e‐03	**3.523e‐02**
CAU.R—DCG.L	3.035	3.841e‐03	**3.841e‐02**
CAU.R—DCG.R	2.917	5.318e‐03	**3.868e‐02**
CAU.R—ROL.R	2.942	4.965e‐03	**3.868e‐02**
*Subtype 1 < subtype 2*			
PCL.L—PAL.R	−5.319	2.567e‐06	**2.054e‐04**
PCL.L—PUT.L	−2.990	4.354e‐03	**3.868e‐02**
MOG.L—AMYG.R	−3.860	3.318e‐04	**8.849e‐03**
MOG.R—ORBinf.R	−3.364	1.499e‐03	**1.999e‐02**

*Note:* The bolded values indicate that the differential edge exhibited a significant difference between the two subtypes (*p*_FDR_ < 0.05).

Abbreviations: AMYG, amygdala; CAU, caudate; DCG, median cingulate and paracingulate gyri; FDR, False Discovery Rate; HES, Heschl gyrus; L, left; MOG, middle occipital gyrus; ORBinf, orbital part of the inferior frontal gyrus; PAL, pallidum; PCL, paracentral lobule; PoCG, postcentral gyrus; PUT, putamen; R, right; ROL, Rolandic operculum; SMG, supramarginal gyrus; TIV, total intracranial volume; VM, vestibular migraine.

### 3.5 Imaging–Clinical Association Findings

In our multivariate regression analyses, two linear regression models and one logistic regression were ultimately retained for interpretation, each containing an imaging feature significantly associated with a clinical variable while controlling for age, sex, education level, and TIV. Specifically, a significant negative association was found between subtype classification (0 = subtype 1 of VM; 1 = subtype 2 of VM) and headache frequency (*β* coefficient, −1.558; 95% confidence interval [CI], −2.785 to −0.332; standard error [SE], 0.610; *p* = 0.014) (Table 2). Additionally, the connectivity between the left PCL (PCL.L) and right PAL (PAL.R) showed a significant negative association with headache frequency (*β* coefficient, −0.522; 95% CI, −0.957 to −0.086; SE, 0.216; *p* = 0.020) and a significant positive association with headache occurrence (*β* coefficient, 1.177; SE, 0.581; Wald chi‐square value, 4.100; odds ratio, 3.245; 95% CI, 1.038 to 10.142; *p* = 0.043) (Table 3). No significant relationships were observed between the remaining imaging features and clinical variables.

**TABLE 2:** Statistical results of imaging–clinical association analyses on the identified neuroanatomical subtypes.

Variables	Association with headache frequency (Linear regression)		
	*B* [95% CI]	SE	*p*
Constant	8.957	4.188	0.037
Age	−0.046 [−0.113, 0.021]	0.033	0.172
Sex (0 = female; 1 = male)	−0.819 [−2.944, 1.306]	1.057	0.442
Education level	−0.207 [−0.362, −0.052]	0.077	0.010
TIV	−0.001 [−0.007, 0.005]	0.003	0.718
Neuroanatomical subtypes (0 = VM I; 1 = VM II)	−1.558 [−2.785, −0.332]	0.610	**0.014**

*Note:* The variance inflation factor of all variables did not exceed 5, which allowed for their inclusion in the model. The bolded value indicates that the imaging feature has a significant *p*‐value (*p* < 0.05).

Abbreviations: B, *β* coefficient; CI, confidence interval; SE, standard error; TIV, total intracranial volume; VM, vestibular migraine; VM I, subtype 1 of VM; VM II, subtype 2 of VM.

**TABLE 3:** Statistical results of imaging–clinical association analyses on the differential structural covariance edges.

Variables	Association with headache frequency (Linear regression)			Association with headache occurrence (Logistic regression)				
	*B* [95% CI]	SE	*p*	*B*	SE	Wald	OR [95% CI]	*p*
Constant	8.500	4.274	0.053	−5.285	14.476	0.133	0.005	0.715
Age	−0.042 [−0.107, 0.024]	0.033	0.206	0.010	0.079	0.016	1.010 [0.865, 1.179]	0.900
Sex (0 = female; 1 = male)	−0.788 [−2.794, 1.218]	0.996	0.433	−1.440	2.154	0.447	0.237 [0.003, 16.158]	0.504
Education level	−0.124 [−0.284, 0.037]	0.080	0.128	−0.488	0.261	3.486	0.614 [0.368, 1.025]	0.062
TIV	−0.003 [−0.008, 0.003]	0.003	0.342	0.010	0.010	1.085	1.010 [0.991, 1.030]	0.298
CAU.R—PoCG.L	−0.003 [−0.321, 0.315]	0.158	0.985	0.797	0.546	2.128	2.218 [0.760, 6.471]	0.145
MOG.R—ORBinf.R	0.240 [−0.222, 0.702]	0.229	0.301	1.696	1.214	1.953	5.452 [0.505, 58.830]	0.162
MOG.L—AMYG.R	−0.280 [−0.706, 0.147]	0.212	0.193	−0.022	0.520	0.002	0.978 [0.353, 2.710]	0.966
PCL.L—PUT.L	−0.092 [−0.682, 0.498]	0.293	0.756	−1.523	0.907	2.820	0.218 [0.037, 1.290]	0.093
PCL.L—PAL.R	−0.522 [−0.957, −0.086]	0.216	**0.020**	1.177	0.581	4.100	3.245 [1.038, 10.142]	**0.043**

*Note:* Variables with a variance inflation factor greater than 5 had been discarded and not included in the models. The bolded values indicate that the imaging feature had significant *p*‐values (*p* < 0.05).

Abbreviations: AMYG, amygdala; B, *β* coefficient; CAU, caudate; CI, confidence interval; L, left; MOG, middle occipital gyrus; OR, odds ratio; ORBinf, orbital part of the inferior frontal gyrus; PAL, pallidum; PCL, paracentral lobule; PoCG, postcentral gyrus; PUT, putamen; R, right; SE, standard error; TIV, total intracranial volume; Wald, Wald chi‐square value.

### 3.6 Functional Implications of the Differential Edges Significantly Different Between Subtypes

With the assistance of BAT, we conducted functional annotation analyses on the differential edges exhibiting significant inter‐subtype differences. As shown in Figure S2, 23 out of 217 functional terms were identified as being related to the differential edges (uncorrected *p* < 0.05), mainly concerned with functions such as perception, emotion, and cognition. The top three functional terms were “gain” (*p* = 0.002), “anticipation” (*p* = 0.007), and “primary somatosensory” (*p* = 0.015), respectively.

### 3.7 Results of Subgroup Analyses

We observed no significant inter‐subgroup differences in the top 80 differential edges across any of the examined subgroups, including sex, migraine/vertigo disease duration (median‐dichotomized), headache occurrence, and history of therapeutic medication use (all *p*_FDR_ > 0.05). Similarly, the proportions of subtype 1/subtype 2 classifications did not differ significantly between subgroups categorized by sex (male, 3/5; female, 16/31; *p* > 0.999, Fisher's exact test), migraine disease duration (shorter, 8/17; longer, 11/19; *p* = 0.717, chi‐square test), vertigo disease duration (shorter, 8/15; longer, 11/21; *p* = 0.975, chi‐square test), headache occurrence (yes, 17/33; no, 2/3; *p* > 0.999, Fisher's exact test), or history of therapeutic medication use (yes, 10/17; no, 9/19; *p* = 0.703, chi‐square test). The findings suggested that both the neuroanatomical features and the identified VM subtypes may be independent of these variables. However, caution is warranted in interpreting these results due to the smaller sample sizes in certain subgroups (especially concerning sex and headache occurrence) and the exploratory nature of the analyses.

### 3.8 Results of Reproducibility Analyses

The two subtypes were consistently identified with different numbers of top differential edges as features. The anatomical distribution of the top 60, 70, and 90 differential edges was similar to that of the top 80 differential edges. The ARI index, which evaluates the consistency of the subtyping results, demonstrated satisfactory agreement across the different strategies. Specifically, the ARI values for each pairwise comparison were: 0.857 for 60–70, 1.000 for 60–80, 0.927 for 60–90, 0.857 for 70–80, 0.789 for 70–90, and 0.927 for 80–90. These results indicate that the subtyping findings were highly consistent and robust to the variation in the number of top differential edges used for clustering.

The differential edges showing significant differences between the two VM subtypes obtained from nonparametric permutation tests were largely consistent with the main findings. Furthermore, all differential edges demonstrating significant inter‐subtype differences in the primary analysis were preserved in the nonparametric permutation test results (Table S1).

## 4 Discussion

The present study explored the individualized structural covariance aberrance in patients with VM using the IDSCN approach. Our study revealed four major findings: (1) Patients with VM exhibited notably large interindividual variations in the number of significantly altered edges, indicating high heterogeneity within the VM population. (2) Despite considerable heterogeneity, patients with VM shared several common connections. (3) Two robust and distinct subtypes of VM were identified based on differential edges, and a link was found between these subtypes and headache frequency. (4) PCL.L–PAL.R connectivity was both associated with headache frequency and occurrence in the VM cohort. These results revealed heterogeneity in VM from a neuroimaging perspective, which might be meaningful for interpreting related symptoms and extending our understanding of the disease.

Clinically, VM is a heterogeneous disorder with substantial variations in clinical manifestations. Despite sharing the same diagnosis, patients with VM exhibit diverse symptom profiles [41, 42, 43]. Heterogeneity has posed challenges to the elucidation of neuropathological mechanisms and has thus affected the identification of robust biomarkers [44]. In this study, using IDSCN analyses, we observed a large interindividual variation of significantly altered structural covariance edges among patients with VM. The results confirmed the clinical heterogeneity in VM and indicated the necessity of employing individualized analytic approaches to investigate VM pathophysiology. However, despite the observed variability across patients, the differential edges identified were mainly distributed among certain regions (i.e., the parietal, subcortical, and cerebellar regions). This distribution remained consistent even when different numbers of top differential edges were selected. This finding indicates that although they presented with high heterogeneity, patients with VM shared several common structural covariance connections.

Based on these differential edges, we further identified two robust and distinct subtypes of VM, which showed an association with headache frequency. In addition, the edges with between‐subtype differences can generally be attributed to migraine‐related neuroimaging evidence, supporting the observed link to headache patterns. The principal finding was that CAU and PCL served as core nodes in the identified subnetwork (with a degree of ≥ 2). CAU constitutes a vital portion of the basal ganglia (BG) structures, which play diverse roles in pain processing, encompassing sensory, emotional/cognitive, and modulatory functions [45]. During migraine attacks, the BG involved in the thalamo‐cortical‐BG loops could participate in the integration of motor, emotional, autonomic, and cognitive responses to pain [46, 47]. Structural and functional alterations of the CAU in migraineurs and their associations with attack frequency have been well documented in previous studies [45, 47]. PCL, a part of the sensorimotor network, is associated with pain perception [48] and pain chronicity [49]. PCL is associated with the occurrence of migraine attacks and their frequency [50]. Overall, combining the clinical basis and brain region functions, these nodes could have appeared in the subnetwork distinguishing the two VM subtypes.

Other nodes in the subnetwork, including temporal (i.e., HES and AMYG), parietal (i.e., SMG and PoCG), occipital (i.e., MOG), frontal (i.e., DCG and ROL), prefrontal (i.e., ORBinf), and subcortical (i.e., PAL and PUT) regions, have also been reported and implicated in the pathophysiology of migraine [45, 51, 52, 53, 54]. The connections among the aforementioned regions distinguished the two VM subtypes linked to headache frequency. Notably, the PCL.L–PAL.R connectivity was both negatively associated with headache frequency and positively associated with headache occurrence in the VM cohort. A possible explanation is that enhanced PCL.L–PAL.R connectivity may reflect more efficient pain processing in the brain that reduces the frequency of headache, while simultaneously increasing sensitivity to headache triggers by making specific neural pathways more prone to activation, which contributes to the occurrence of headache. The association between PCL.L–PAL.R and pain processing is supported by a previous study employing Granger causality analysis, which revealed that the neural activity of the PAL.R could predict activity of the PCL.L in patients with attention‐deficit/hyperactivity disorder, interpreted to be related to somatosensory dysfunctions including pain sensation [55]. Thus, we further assumed that the observed structural covariance between PCL.L and PAL.R might be a meaningful neuroimaging marker for headache in patients with VM.

Regarding the potential deeper functional implications of these differential edges showing inter‐subtype differences, we identified 23 associated functional terms, primarily related to perception, emotion, and cognition, with the top three significant being “gain”, “anticipation”, and “primary somatosensory”. Besides the more straightforward concept of “primary somatosensory”, the term “gain” likely reflects sensory gain (i.e., hyperalgesia) provoked by pain‐induced somatosensory plasticity [56], resulting in heightened sensitivity to pain stimuli. Additionally, it may also represent reward‐like responses associated with pain alleviation during periods of pain relief [57]. The significant association with “anticipation” suggests that the differential edges may be involved in interoceptive predictions in the brain [58], potentially linked to the anticipation of migraine attacks. This is reinforced by evidence indicating an association between migraine frequency and heightened pain anticipation in migraineurs [59]. Taken together, the findings support the potential involvement of differential edges in pain processing mechanisms.

The variable relationship between headaches and VM attacks is frequently observed in clinical practice. Headaches may or may not be present during VM episodes and are often less severe than migraine‐related headaches [43]. In our study, the association between the identified subtypes and headache frequency coincided with the clinical basis of the disease, indicating that heterogeneity in headache frequency is an intrinsic feature of VM. The findings partially explained the conflicting structural aberrances revealed by group‐level differences in traditional case–control studies on VM, highlighting the importance of subtyping analyses. Our study supports the hypothesis that VM could be a multifactorial disorder with nonunique pathophysiology, i.e., different phenotypes may be linked to a distinct pathophysiological basis [2]. This may offer new perspectives on the taxonomy of VM and be instructive for precise diagnosis and individualized treatment of the disease in clinical settings [1].

Our study had several limitations. First, given that this is an exploratory investigation with a relatively small sample size, further research involving a larger cohort is imperative to confirm the current findings. Second, since the participants were recruited from a single center, the generalizability of the results must be further assessed through validation with a multicenter dataset. Third, although an association between subtypes and headache frequency was found, it cannot be considered specific, as other vertigo characteristics and accompanying symptoms could not be analyzed due to our incomplete data on these variables. Future studies should examine the relationships between neuroanatomical subtypes and finer clinical indicators, which they may exist. Fourth, the IDSCN method relies on the reference network of the control group, and potential heterogeneity within this group, such as subclinical brain changes, may influence the results. If future advancements in methodology address this, the issue of heterogeneity within the entire study cohort will also warrant further validation. Fifth, the study design was cross‐sectional, which limits the ability to infer causal relationships between individualized structural alterations and disease progression or treatment effects. In the future, prospective longitudinal studies tracking the dynamic changes are warranted to resolve these issues. Finally, our study did not include a comparison with other types of migraines, such as migraine without aura. Future research that incorporates this comparison, exploring both shared and distinct brain network abnormalities across different types of migraines (as seen in an IDSCN study on mental disorders [60]), will be intriguing and could provide a deeper insight into the common features across migraine forms as well as the unique signatures specific to each type.

In conclusion, this study revealed considerable heterogeneity of structural covariance abnormalities in patients with VM and identified two neuroanatomical subtypes associated with headache frequency. These findings would improve our current understanding of this disease in terms of individual differences and may facilitate potential clues to precise diagnosis and individualized treatment of VM.

## Author Contributions

W.C., J.K., and C.H. designed the study. H.Z., X.X., and Q.F. collected the data. W.C. and L.D. analyzed the data. W.C. prepared the figures and drafted the manuscript. J.K. and C.H. edited and revised the manuscript. C.H. supervised the project. All authors reviewed and approved the final manuscript.

## Ethics Statement

This research was conducted in accordance with the Declaration of Helsinki and was approved by the Ethics Committee of the First Affiliated Hospital of Soochow University. Written informed consents were achieved from all subjects prior to their participation.

## Conflicts of Interest

The authors declare no conflicts of interest.

## Supporting information


**Data S1:** cns70599‐sup‐0001‐Supinfo.docx.

## Data Availability

The data supporting the findings of this study are available from the corresponding author upon reasonable request.
