# Supplementary material for: Exploring Heterogeneity in Vestibular Migraine Using Individualized Differential Structural Covariance Network Analysis
Source: CNS Neurosci Ther. 2025 Sep 8;31(9):e70599. doi: 10.1111/cns.70599 (PMC12415590; doi:10.1111/cns.70599)
Supplement: Supplementary file 1 — Data S1: cns70599‐sup‐0001‐Supinfo.docx. [file CNS-31-e70599-s001.docx]

**Supplementary Material**

**Table S1.** Statistical results of differential edges showing significant differences between the two VM subtypes based on nonparametric permutation tests with 10,000 iterations (controlling for sex, age, education level, and TIV)

| **Edge/condition** | **Mean difference** | **P** | **P_FDR_** |
| --- | --- | --- | --- |
| **subtype 1 > subtype 2** |  |  |  |
| CAU.L – HES.R | 1.085 | 0.0003 | **4.800e-03** |
| CAU.L – ROL.R | 1.140 | 0.0081 | **4.985e-02** |
| CAU.R – HES.R | 1.147 | 0.0001 | **2.667e-03** |
| CAU.R – SMG.L | 1.186 | 0.0002 | **4.000e-03** |
| CAU.R – PoCG.L | 1.557 | 0.0011 | **1.257e-02** |
| CAU.R – PoCG.R | 1.020 | 0.0077 | **4.985e-02** |
| CAU.R – DCG.L | 1.432 | 0.0015 | **1.500e-02** |
| CAU.R – DCG.R | 1.443 | 0.0027 | **2.160e-02** |
| CAU.R – ROL.R | 1.178 | 0.0032 | **2.327e-02** |
| **subtype 1 < subtype 2** |  |  |  |
| PCL.L – PAL.R | -2.422 | < 0.0001 | **< 2.667e-03** |
| PCL.L – PUT.L | -1.105 | 0.0023 | **2.044e-02** |
| MOG.L – AMYG.R | -1.360 | 0.0001 | **2.667e-03** |
| MOG.R – ORBinf.R | -1.167 | 0.0005 | **6.667e-03** |

The bolded values indicate that the differential edge exhibited a significant difference between the two subtypes (P_FDR_ < 0.05).

VM, vestibular migraine; TIV, total intracranial volume; FDR, False Discovery Rate; L, left; R, right; CAU, caudate; HES, Heschl gyrus; ROL, Rolandic operculum; SMG, supramarginal gyrus; PoCG, postcentral gyrus; DCG, median cingulate and paracingulate gyri; PCL, paracentral lobule; PAL, pallidum; PUT, putamen; MOG, middle occipital gyrus; AMYG, amygdala; ORBinf, orbital part of the inferior frontal gyrus.

**
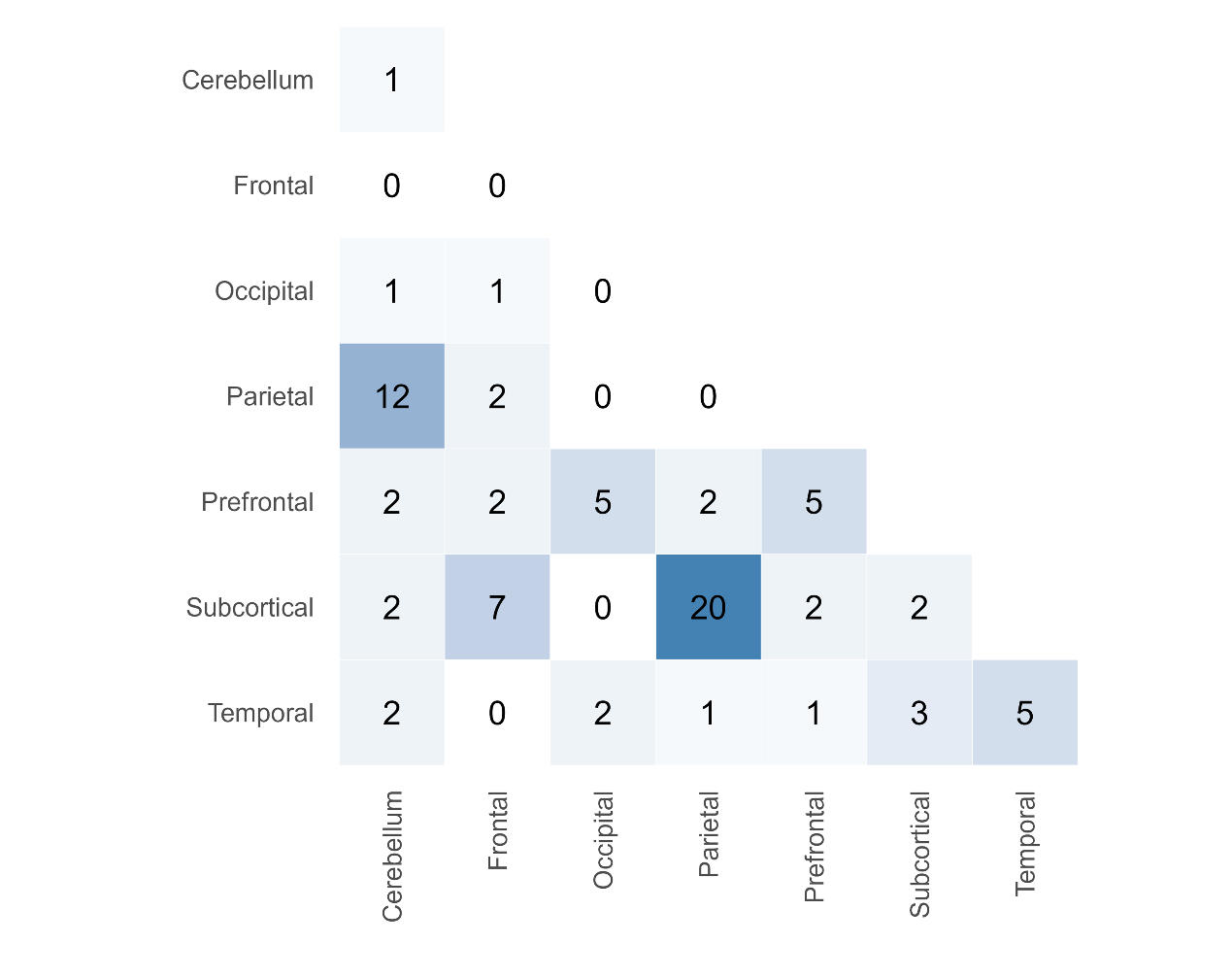
**

**Fig. S1.** Distribution of the top 80 differential edges across patients with VM. The number of edges between and within areas (defined based on the AAL116 atlas) is presented. These edges were distributed mainly between the parietal lobe and subcortical regions as well as between the parietal lobe and cerebellum.

VM, vestibular migraine; AAL, Automatic Anatomical Labeling

**
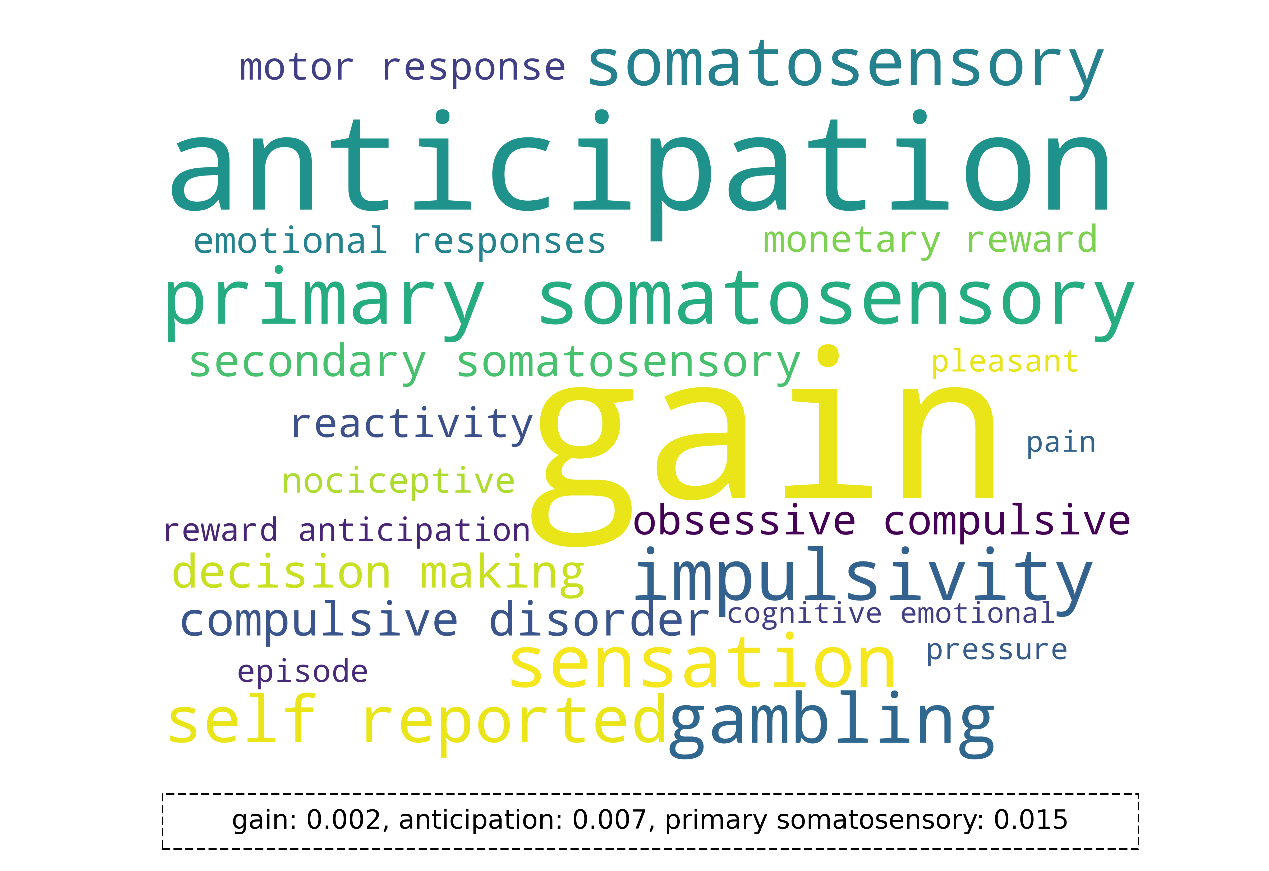
**

**Fig. S2.** Word clouds representing the 23 significant functional terms (uncorrected P < 0.05) identified through the functional annotation of differential edges with significant inter-subtype differences. The size of each term reflects its significance, with larger text indicating a smaller P value. The dashed box at the bottom indicates the P values of the top three most significant terms.
